# Formal Specification and Validation of a Hybrid Connectivity Restoration Algorithm for Wireless Sensor and Actor Networks ^†^

**DOI:** 10.3390/s120911754

**Published:** 2012-08-29

**Authors:** Muhammad Imran, Nazir Ahmad Zafar

**Affiliations:** 1 Deanship of E-Transactions and Communication, King Saud University, Riyadh, Saudi Arabia; 2 Department of Computer Science, King Faisal University, Hofuf, Saudi Arabia; E-Mail: nazafar@kfu.edu.sa

**Keywords:** WSANs, connectivity restoration, controlled and coordinated actor relocation, formal specification, Z notation

## Abstract

Maintaining inter-actor connectivity is extremely crucial in mission-critical applications of Wireless Sensor and Actor Networks (WSANs), as actors have to quickly plan optimal coordinated responses to detected events. Failure of a critical actor partitions the inter-actor network into disjoint segments besides leaving a coverage hole, and thus hinders the network operation. This paper presents a **P**artitioning detection and **C**onnectivity **R**estoration (PCR) algorithm to tolerate critical actor failure. As part of pre-failure planning, PCR determines critical/non-critical actors based on localized information and designates each critical node with an appropriate backup (preferably non-critical). The pre-designated backup detects the failure of its primary actor and initiates a post-failure recovery process that may involve coordinated multi-actor relocation. To prove the correctness, we construct a formal specification of PCR using Z notation. We model WSAN topology as a dynamic graph and transform PCR to corresponding formal specification using Z notation. Formal specification is analyzed and validated using the Z Eves tool. Moreover, we simulate the specification to quantitatively analyze the efficiency of PCR. Simulation results confirm the effectiveness of PCR and the results shown that it outperforms contemporary schemes found in the literature.

## 1. Introduction

**Figure 1.**
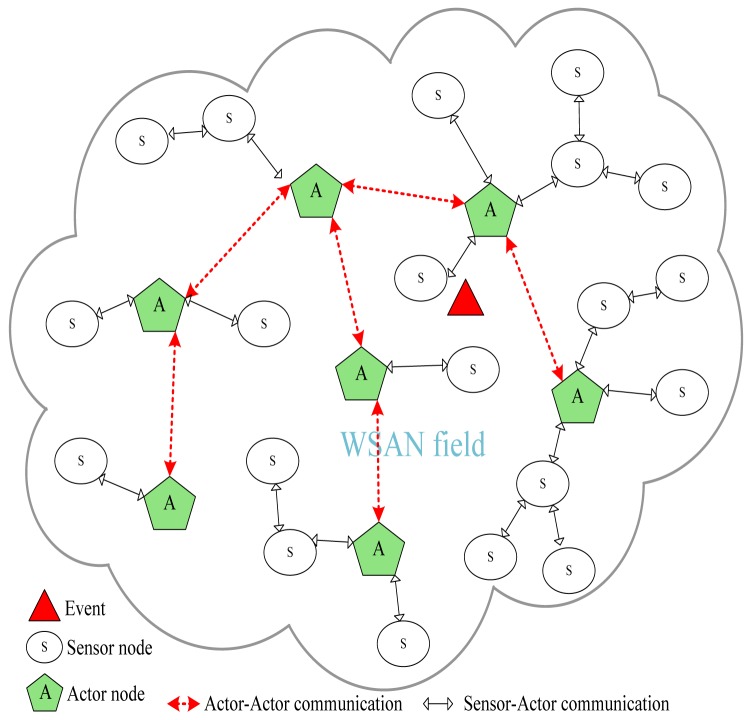
An example of autonomous wireless sensor and actor network setup.

Wireless sensor and actor networks (WSANs) are envisaged to be a promising technology for mission-critical applications that require autonomous and intelligent interaction with the environment. Examples of such applications include fire detection and containment, disaster management, urban search and rescue (USAR), homeland security, battlefield surveillance, space exploration and nuclear, biological and chemical attack detection and prevention. These applications are also regarded as safety-critical applications because an inappropriate action may have catastrophic consequences such as loss of human life, severe injuries, large-scale environmental damage and considerable economical penalties. In these critical applications, WSANs employ a number of sensor nodes that report events of interest to one or multiple actors [1]. The concerned actors receive event notifications, process them and share with peer actors to effectively respond to events such as fires, earthquakes and disasters. Figure 1 illustrates an example of an autonomous WSAN environment.

The inherent characteristics of a WSAN require actors to collaborate and coordinate with each other in planning an optimal response and synchronize their operations. For example, in USAR, sensors and actors are deployed in an area damaged by an event such as a fire, earthquake or other disaster. The sensors detect the presence of survivors in the vicinity and report it to the actors. The actors equipped with necessary life support equipment receive the sensors data, process it and share it with peer actors to identify the most appropriate set of actors. These actors are responsible for rescuing survivors immediately or provide them with life saving necessities such as water, oxygen or even some sort of medicine for a short period until the rescue team arrives. The role of actors is extremely crucial for a timely response to this type of events in order to prevent serious consequences. This requires actors to interact with each other and determine the most appropriate set of actors that will participate in the operation. To enable such interaction, actors must establish and maintain the inter-actor topology that serves as a backbone of the network.

The requirement for a fully autonomous WSAN has been realized due to several incidents that cost unbearable lost of lives in situations too difficult or dangerous for humans to operate. For example, during the 9/11 rescue operations, four hundred and seventy-nine rescue workers lost their lives in making the evacuation a success. Similarly, actors operating in hazardous and inhospitable environments are subject to failures. Failure of a critical actor partitions the inter-actor network into disjoint segments besides leaving uncovered regions. This may stop inter-actor interactions altogether and the network becomes incapable of delivering a timely response to serious events. The autonomous, unattended and resource restricted nature of WSAN necessitates self-healing and agile recovery processes that involve reconfiguring the inter-actor topology with minimal overhead. Moreover, criticality of the applications, distributed, dynamic and complex operation of the network demand for rigorous and reliable validation of recovery schemes.

Most of the published connectivity restoration schemes are purely reactive [2–5], triggering the recovery process once the failure of a node is detected. Reactive schemes may not be suitable for mission-critical time-sensitive applications due to procrastination. Moreover, existing recovery schemes lack rigorous validation and entirely rely on non-formal validation *i.e.*, simulation. Simulation-based results are useful only for statistical performance analysis and do not guarantee the correctness of the approach that is crucial in safety-critical applications. Therefore, we advocate the use of complementary validation techniques for connectivity restoration algorithms, especially in safety-critical applications.

Formal methods are advanced mathematics-based techniques, having computer tool support, used for modeling and formal specification of complex and critical systems. Z notation [6] is a model-centered approach based on sets, sequences, bags, relations, functions and predicate logic which is used at an abstract level of specification of systems. Z can be used for specifying behavior of distributed as well as sequential programs because of its accommodation of abstract data types.

This paper presents a **P**artitioning detection and **C**onnectivity **R**estoration (PCR) algorithm that proactively identifies critical/non-critical nodes and rapidly repairs the topology in case of critical node failure. Each actor determines whether it is critical or not based on localized information and designates a suitable neighbor as a backup that continuously monitors and triggers a recovery in case of primary failure. The algorithm is recursively executed until all actors become connected. We use formal and non-formal techniques for correctness and performance validation. We model a WSAN network as a dynamic graph and transform PCR into a corresponding formal specification using Z notation. This formal specification is analyzed and validated using the Z Eves tool [7]. Moreover, the performance of PCR is validated through extensive simulations. The simulation results confirm the effectiveness of PCR and the results are shown to outperform contemporary schemes found in the literature.

It is worth noting that our algorithm does not require additional actors, despite providing adequate redundancy. To the best of our knowledge, this is the first effort that employs formal (formal specification) and non-formal (simulation) techniques for rigorous validation of recovery schemes. It is worth mentioning that our algorithm is equally applicable for mobile sensor networks and mobile robot networks.

This paper is organized as follows: Section 2 discusses the system model and problem statement. The related work is discussed in Section 3. The proposed PCR algorithm is detailed in Section 4. Section 5 presents the formal specification and analysis of PCR. The performance evaluation of PCR is presented in Section 6. Section 7 concludes the paper.

## 2. System Model and Problem Statement

Our algorithm is applicable to WSANs that involve sensors and actors. Sensors are inexpensive and have scarce resources, whereas actors are more powerful nodes in terms of energy, communication and computation power (processing and memory). The communication range of an actor refers to the maximum Euclidean distance that its radio can reach and is assumed to be larger than that of sensors. Both sensors and actors are deployed randomly in an area of interest. After deployment, actors are assumed to discover each other and form a connected inter-actor network. An actor is assumed to be able to move on demand and is aware of the positions of its 1-hop neighbors.

**Figure 2.**
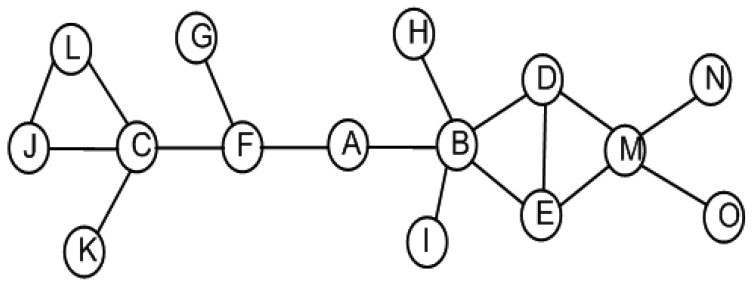
Graphic representation of a connected inter-actor network.

The impact of an actor's failure depends on the position of that actor in the network topology. For example, losing a leaf/non-critical node, such as *K* or *D* in Figure 2, does not affect inter-actor connectivity. Meanwhile, the failure of a critical node such as *F* partitions the network into disjoint segments. In order to tolerate critical node failure, three approaches are identified: (i) proactive; (ii) reactive and (iii) hybrid. Proactive approaches establish and maintain bi-connected topology in order to provide fault tolerance. This necessitates a large actor count that leads to higher cost and becomes impractical. On the other hand, in reactive approaches the network responds only when a failure occurs. Therefore, reactive approaches might not be suitable for mission-critical time-sensitive applications. In hybrid approaches, each critical actor proactively designates another appropriate actor to handle its failure when such a contingency arises in the future. We argue that a hybrid approach will better suit autonomous WSANs that are deployed for mission-critical time-sensitive applications due to the reduced recovery time and overhead.

Despite the fact that sensor networks have attracted considerable attention from the research community in recent years, however, “we are still nowhere near the production of industrial grade WSAN software that can be relied upon for mission-critical applications” [8]. The success or performance of WSANs in these applications is strongly dependent on the correctness of the algorithms/protocols running on the underlying network. Therefore, the criticality of the applications, distributed, dynamic and complex operation of the network necessitates for rigorous and reliable validation of recovery schemes.

## 3. Related Work

### 3.1. Connectivity Restoration

The issue of fault tolerance in different contexts for WSANs has only been studied in few publications. For instance, the fault-tolerant model presented in [9] assigns multiple actors to each sensor and multiple sensors to each actor in order to ensure guaranteed event notification, even in cases of either failure or inaccessibility. However, our fault-tolerant model operates in the context of maintaining inter-actor connectivity rather than reliable sensor-actor communication. Few researchers have exploited node mobility as a means for performance optimization both in sensor networks and WSANs. However, exploiting node mobility to mend severed topologies has just recently started to attract attention. The reader is referred to [10] for a comprehensive survey of node relocation strategies.

The existing work can be categorized into block and cascaded movement. Block movement often requires a high pre-failure connectivity in order for the nodes to coordinate their response. An example of block movement based approaches is reported in [11], where the initial network is assumed to be 2-connected and goal is to sustain such 2-connectivity even under link or node failure. The idea of movement of robots is similar to ours but their approach requires a centralized algorithm. Das *et al.* presented a distributed approach to the similar problem that strives to restore 2-connectivity in [12]. Unlike [11] and [12], our algorithm focuses on providing 1-connectivity.

Block movements often become infeasible in the absence of a higher level of connectivity. Therefore, few researchers have pursued cascaded node movement that can be further categorized based on network state information that nodes are assumed to maintain. Some approaches like DARA [2], or PADRA [13] require each actor to maintain 2-hop neighbors. Others, such as RIM [3], C^3^R [4], and VCR [5] avoid the increased overhead for tracking 2-hop neighbors and require each node to maintain only its directly reachable nodes, *i.e.*, 1-hop neighbors. Like our proposed PCR algorithm, DARA strives to restore connectivity lost due to cut-vertex failures. However, DARA requires more network state in order to ensure convergence. Meanwhile, in PADRA, Akkaya *et al.* identify a connected dominating set (CDS) of the whole network in order to detect cut-vertices. Since the CDS based method is not accurate for critical node detection, they perform a depth-first search (DFS) on each member for the CDS to confirm whether that the node is really a cut vertex or not. Although, they use a distributed algorithm their solution still requires 2-hop neighbors' information that increases messaging overhead. Another work proposed in [14] also uses 2-hop information to detect cut-vertices. The proposed PCR algorithm relies only on 1-hop information and reduces the communication overhead. Although RIM, C^3^R and VCR use 1-hop neighbor information to restore connectivity, they are purely reactive and do not differentiate between critical and non-critical nodes, whereas, PCR is a hybrid algorithm that proactively identifies critical nodes and designates them an appropriate backup that is responsible for restoring lost connectivity.

### 3.2. Formal Methods

Formal methods are mathematics-based techniques used for describing properties of software and hardware systems [15]. The Z notation is a model-centered approach usually used for specifying the behavior of programs by abstract data types and has standard set operators. The Z allows organizing a system into its smaller components using a powerful data structure named schema. The schema defines a way in which state of a system can be specified and further refined by describing details of a system [16]. A schema has two parts, one for variables definitions and another for defining the properties of these variables.

To the best of our knowledge, the only effort to model wireless sensor and actuator networks has been reported in [17]. They used Colored Petri Nets (CPNs) to model and validate some components of sensor and actuator networks. However, it is unclear that the CPN approach can be used to model more sophisticated WSANs algorithms like PCR. Recently, some works in the literature [18–20] have advocated the use of formal methods in *ad hoc* and sensor network protocol validation. For example, Maag *et al.* have proposed a formal specification based conformance testing methodology to validate the routing protocols in MANET, especially DSR [18], while the authors in [19] used Real-Time Maude for modeling, performance estimation and model checking of the state-of-the-art OGDC algorithm for sensor networks. A comprehensive survey of formal and informal techniques for protocol validation in *ad hoc* and sensor networks is presented in [20]. Some other similar work on a formal specification of MANET routing protocols can be found in [21–23].

Our work is different from the formal method-based approaches discussed above and others in that: (i) we model WSAN as a dynamic graph and transform PCR into a corresponding formal specification using Z notation. This formal specification is analyzed and validated using the Z Eves tool, and (ii) we simulate the specification to analyze the effectiveness and efficiency of PCR.

## 4. Partitioning Detection and Connectivity Restoration

As stated earlier, hybrid algorithms better suit mission-critical time-sensitive applications that require a rapid recovery. The proposed PCR algorithm is hybrid in the sense it consists of two parts *i.e.*, pre-failure planning and post-failure recovery.

**Figure 3.**
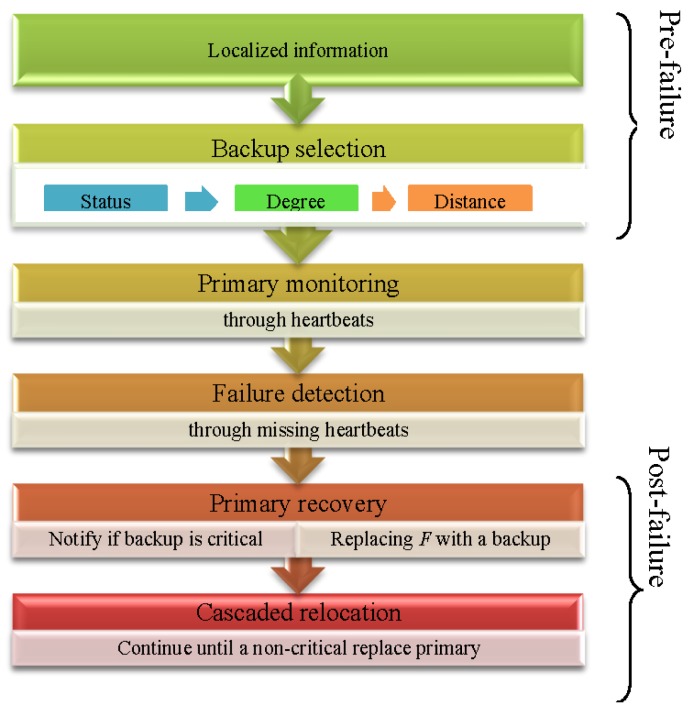
Overview of the PCR algorithm.

Figure 3 shows the overall working of the PCR algorithm. In the pre-failure planning, PCR identifies critical/non-critical actors (primary) based on localized information and designates an appropriate backup for each critical node. The backup starts monitoring its primary and detects the failure through missing heartbeats. In the post-failure recovery, a pre-designated backup if critical notifies it's backup and moves to the location of the primary. This cascaded relocation continues until a non-critical replaces the primary. The detailed algorithm is described in the sections that follow.

### 4.1. Pre-Failure Planning

Failure of a critical actor disconnects the neighbors and they are unable to coordinate because they have limited network information. Therefore, PCR pursues pre-failure planning to identify critical actors and designate them an appropriate backup.

#### Distinguishing critical/non-critical actors

**Figure 4.**
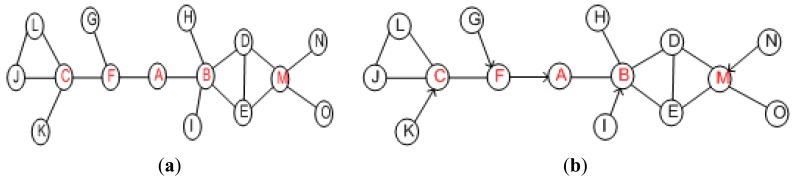
Determine critical nodes and assign backup: (**a**) 1-hop critical/non-critical and (**b**) designate backup using PCR.

As stated above, the failure of critical actor partitions the inter-actor network into disjoint segments. The absence of a non-critical node does not affect the connectivity. PCR opts to distinguish between critical/non-critical nodes and designate a backup for each of these critical actors. The existing algorithms to determine cut-vertices in a graph can be categorized into centralized and distributed. Centralized algorithms [24,25] may not be suitable for large scale dynamic networks due to the fact they involve huge communication overhead in maintaining network state information. Frequent changes in the WSAN topology favors distributed and highly localized algorithms. Some localized algorithms such as [26] require only 1-hop neighbors' positional information at the expense of lower accuracy of cut-vertices identification. Basically, some nodes are marked as critical while they are not cut-vertices globally. However, no critical node will be missed and the accuracy of determining non-critical ones is 100%. The fact that PCR prefers to designate a non-critical backup, such a category of approaches fits well and the reduced accuracy is not a major concern. Therefore, PCR employs a simple localized cut-vertex detection procedure that only requires 1-hop positional information to detect critical/non-critical nodes. The procedure is based on [26] and runs on each node in a distributed manner. An actor is critical if its immediate neighbors become disconnected without it, non-critical otherwise. Leaf nodes are also determined as non-critical. For instance, Figure 4(a) shows the 1-hop critical/non-critical nodes. The details of the procedure are provided in [27].

#### Backup selection

The critical actors choose and designate an appropriate backup among neighbors once the critical/non-critical nodes are differentiated. Several criteria can be defined when choosing a backup, depending on the application-level interests. The selection of a backup among 1-hop neighbors is based on the following ordered criteria:

1. *Neighbor Actor Status* (*NAS*): Each critical actor prefer to designate a non-critical node as backup since moving such a node will not affect inter-actor connectivity. Moreover, this will restrict the scope of recovery and reduce movement overhead.
2. *Actor Degree* (*AD*): The number of neighbors reflects the actor degree. In case a non-critical node in the neighborhood is not available, PCR prefers to designate a least degree critical node as backup since few nodes will lose direct communication links to that backup when it moves. However, moving a critical node may trigger a series of cascaded relocations that we will discuss later in this section.
3. *Inter-actor Distance* (*ID*): A nearby neighbor is preferred in case multiple neighbors have the same AD. This will help to shorten the recovery time and reduce the movement overhead that is crucial for resource-constrained mission-critical applications.

An actor may be selected as backup for more than one node. A primary node selects another backup using the same procedure specified above in case a backup fails or moves outside the range of its primary. Since the backup selection criterion is based on 1-hop information, therefore, it may not always lead to an optimal solution. However, being highly localized helps PCR scale for large networks. Figure 4(b) shows the setup where each critical actor designates another as backup where the arrowhead points towards the primary. Note that PCR provides redundancy without requiring extra resources in terms of actors. It employs existing actors just to take care of each other. One may argue that PCR imposes additional overhead in the form of pre-failure planning, *i.e.*, maintaining backup. In the pre-failure planning, PCR exploits the status update messages that actors exchange as part of their normal network operation. Therefore, PCR mainly involves computation overhead in terms of selecting and maintaining a backup. Since actors are powerful nodes, therefore, computation is not a major concern since it is far less energy-demanding than messaging and node movement. Nevertheless, PCR strives to limit this computation overhead to critical actors only.

### 4.2. Primary Monitoring and Failure Detection

Once each critical actor selects an appropriate backup, it is notified through regular heartbeat messages. The pre-designated backup starts monitoring its primary through heartbeats. Missing a number of successive heartbeats at backup indicates the failure of the primary. After failure detection, the backup triggers the post-failure recovery process as detailed in the following section.

### 4.3. Post-Failure Recovery

**Figure 5.**
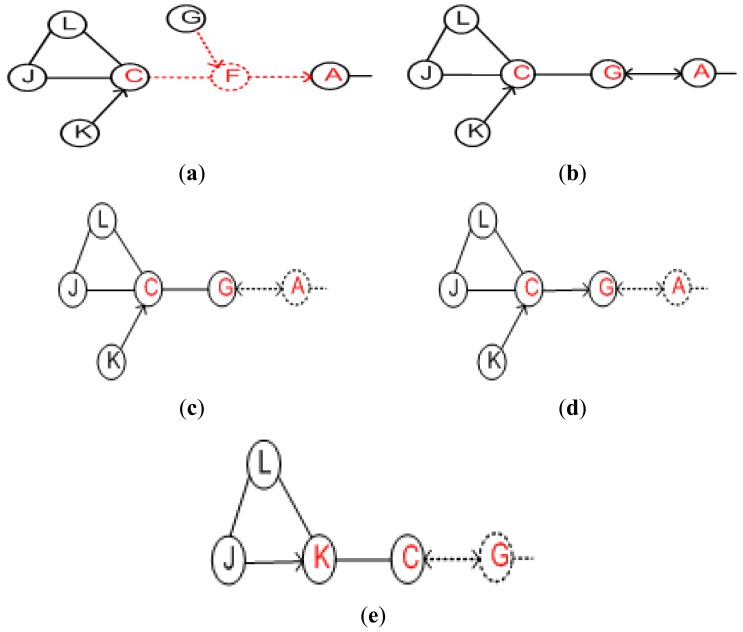
Applying the recovery process of PCR.

The post-failure recovery process is initiated by the pre-designated backup upon failure detection. The scope of recovery depends on the NAS. If the backup is a non-critical actor then it simply replaces the primary and the recovery would be complete. However, if the backup is also a critical node then a cascaded relocation is performed. Basically, repositioning of actor *A_i_* in response to the failure of *A_f_* will be interpreted by its backup *A_j_* as if *A_i_* is lost and *A_j_* will thus move to replace *A_i_*. The detail of the recovery process is as follows:

The pre-assigned backup immediately triggers a recovery process once it detects failure of its primary. The status of backup determines the scope of recovery which can be among the following three scenarios. First, if a backup is a non-critical node the scope of recovery will be limited because it does not require further relocations. The backup actor moves to the location of the failed primary and exchanges heartbeat messages with its new neighbors. It selects and designates a new backup since it has become a critical node at the new position. This movement alerts the other primary nodes (if any) at the previous location to choose a new backup for themselves. An illustrative example is provided in Figure 5(a), where non-critical backup *G* simply replaces its primary (*i.e., F*) and selects a backup for itself. The second scenario is when the failed (primary) and backup node are both critical nodes and simultaneously serving as backup for each other. This scenario is articulated in Figure 5(b). Actor *G* is serving as backup of another actor *A* and *vice versa* as shown in Figure 5(b). The actor *G* detects failure of *A* and selects another actor “*C*” as backup, as shown in Figure 5(d). Then *G* moves to the position of *A*. The newly assigned backup actor performs a cascaded relocation as discussed below and is shown in Figure 5(e), with *G* replacing *A, C* replacing *G* and *K* replacing *C*. The third scenario is when the backup is a critical node. In this case, the backup actor will notify its own backup so that the network stays connected. This scenario may trigger a series of cascaded repositioning of nodes as explained above.

### 4.4. Pseudo Code of PCR Algorithm

**Figure 6.**
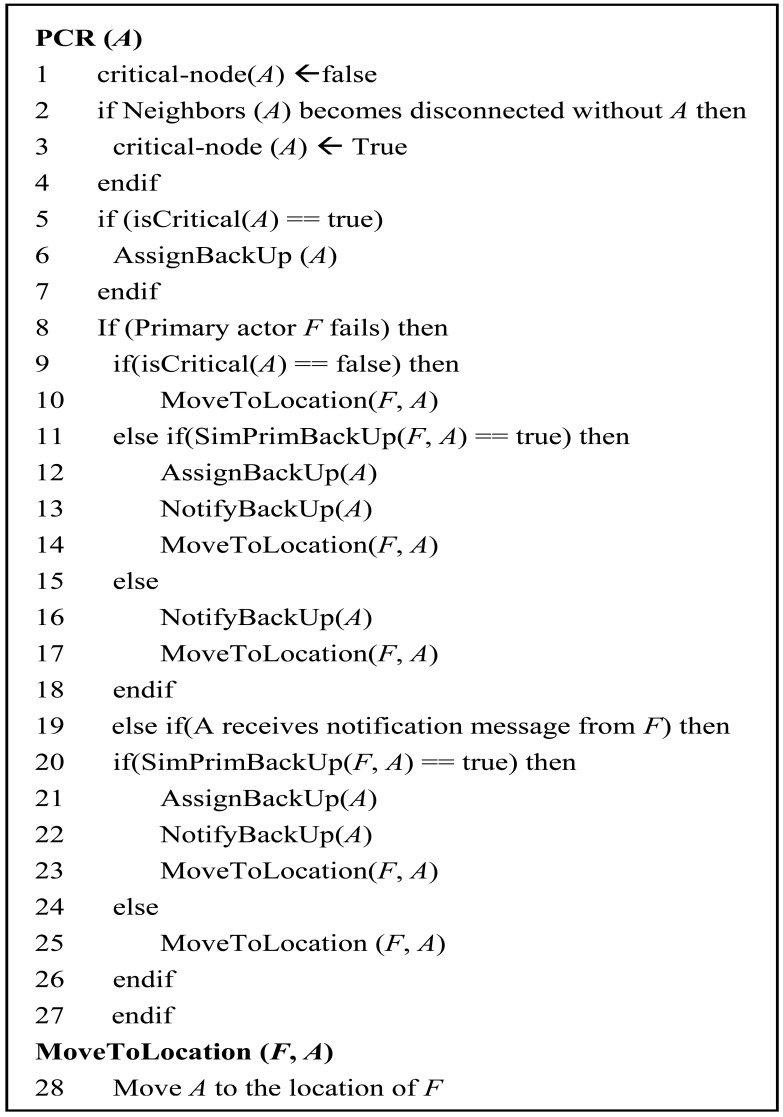
High level pseudo code for PCR algorithm.

Figure 6 shows the high level pseudo code of PCR algorithm running on each actor in a distributed manner. Initially, all the actors are initialized as non-critical (line 1). A localized cut-vertex detection procedure determines whether node *A* is critical or not (lines 2–4). If actor *A* is critical then it will select and designate an appropriate backup actor among the neighbors (lines 5–7). The selection of backup is made based on the criteria specified in Section 4. The backup actor *A* detects failure of its primary by continuously monitoring its health through heartbeat messages. Upon detecting the failure of the primary, it initiates the recovery process. If backup actor *A* is non-critical, it simply moves to the location of *F* (lines 9–10). If node *A* is critical and simultaneously primary and backup of node *F* (*i.e.*, SimPrimBackUp) then it selects another node as backup. In other words, node *A* and *F* were serving as backups for each other, Since when *F* fails, node *A* loses not only its primary but also its backup, therefore node *A* appoints another backup before going to replace *F*. Node *A* notifies its movement to newly assigned backup and moves to the location of *F* (lines 11–14). Otherwise, if node *A* is a critical node it notifies its backup and moves to replace *F* (lines 15–18). If node *A* receives a movement notification message from *F*, it checks whether *F* is serving as both primary and backup. If so then node *A* designates a new backup, notifies its neighbors and moves to replace *F* (lines 19–23), otherwise, it moves to replace *F* (lines 24–27).

## 5. Formal Specification and Analysis

In this section, reasoning of describing formal specification of complex systems is provided. An introduction and critical analysis to Z notation is also given. Finally, the suitability of applying Z notation in modeling of wireless sensor and actor networks is argued.

### 5.1. Reasoning of Using Formal Description

There exist various traditional tools and techniques which are typically used for expressing the properties of software systems, however, these methods require a full commitment because the specification produced must be used to construct a complete and consistent model which will be assumed as a baseline for the further development. For complex and incomplete models such methods are not effective for a complete validation and verification of a large scale software specification. Consequently, it needs to apply mathematics-based techniques to overcome the weaknesses of these traditional approaches. Formal methods are advanced mathematics-based techniques, having computer tool support, used for modeling and formal specification of complex and critical systems. Formal specification of a system requires a detailed and thorough study of the design. Although it cannot be proved that formal specification of a system is correct, in general, because initially requirements are always provided in an informal way, but, it can be used to prove the properties and consistency in the design enhancing confidence to develop the systems. Experience of applying formal methods [28] has shown that it is an effective way of modeling, specifying, analyzing and verifying of properties of complex systems.

### 5.2. Z Notation

Formal methods are notations based on discrete mathematics such as logic, set theory, graph theory, automata and algebraic systems having sufficient computer tool support used for describing and analyzing properties of software and hardware systems. Formal methods may be classified as property oriented or model descriptive. Property-based methods are used to describe software in terms of properties and invariants. The property-based specification languages are more abstract but are also executable. Model-oriented methods are used to construct models of a system emphasizing both the statics and dynamics of the system. There is a trade-off using model-oriented and property-based specification languages.

Z notation is a model-centered approach based on sets, sequences, bags, relations, functions and predicate logic [16] which is used at an abstract level of specification of systems. Z can be used for specifying the behavior of distributed as well as sequential programs because of its abstract data types. The Z has standard set operators, for example, union, intersection, comprehensions, Cartesian products and power sets.

The Z notation is applied in this research because it is a model-oriented approach having rigorous characteristics and is model-based on first order logic with set theory and, consequently, is powerful and very expressive, which is one of the requirements in selecting any specification language for modeling of distributed systems. Moreover, Z is based on the standard mathematical notations used for the specification of abstract properties unlike a detailed description language. Further, the Z notation allows organizing a system into its smaller components using a powerful data structure named schema. The schema defines a way in which state of a system can be described and refined. Refinement is a promising way of Z supporting verifiable transformation from an abstract specification into an executable code. The use of schema structures helped us to reduce the complexity because of its abstract and re-useable characteristics. Formal specification described using Z notation can further be refined and transformed to an implemented system. In this way, we can claim that Z reduces the complexity of systems by abstraction and structuring power, and eliminates unnecessary details. Finally, development from abstraction to detailed analysis made it easy to propose a simple and understandable model ready to be used for simulation.

### 5.3. An Integration of Graph Theory and Z Notation

The applications of graph theory are becoming increasingly significant in all areas of computer science and engineering. Particularly, communication and network systems cannot be analyzed and optimized without using the structures and algorithms of graph theory. This is because the origin of graphs is based on describing networks, their properties and encodings. More important is that theory of graphs has been enriched in last couple of decades, particularly in the area of computer science, and its applications are extended to social and complex networks. As the Z method is based on set theory and first order predicate logic as mentioned above, the sets and relations are the fundamentals of describing composite types in Z notation. On the other hand, the structures of graphs are same that are defining collections of objects and then establishing links or relations over the objects. Hence, any model in graph theory can easily be transformed into Z notation for further analysis and proof generation using computer tools. That is why, initially, the wireless sensor and actor network is described using graph structures and then transformed to Z notation and an algorithm is proposed for network recovery in case of any failure in the system.

### 5.4. Formal Specification of PCR Algorithm

In this section, a formal specification of the proposed failure recovery algorithm for wireless sensor and actor network is described using Z. Formal analysis of the specification is provided using the Z/Eves toolset in the next section. First of all, topology under the wireless sensor and actor network is described, which must be maintained throughout the life of the network. The topology consists of a set of nodes and a set of edges. The set of nodes is a power set of *Node* where *Node* is assumed as a set at an abstract level of specification in Z notation. In modeling using sets in Z, a high level of abstraction is supposed. For example, we do not define any effective procedure for deciding whether an arbitrary element is a member of the abstract set. Consequently, *Node* defined below is a set over which operators of set theory cannot be defined. For example, cardinality to know the number of elements in the *Node* cannot be defined. Similarly, the subset, union, intersection and complement operators are not well-defined as well. To define such operations new set must be created based on the abstract set. The set of edges of the topology is a relation over the collection of the nodes. The topology schema is denoted by *Topology* which consists of two variables *nodes* and *edges* contained in the first part of the schema. The invariants over the topology are defined in the second part of it:

[*Node*] *Topology**nodes:*

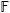

*Node**edges:*

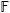
 (*Node* × *Node*)∀*n: Node* | *n* ∈ *nodes* • ∃*e: Node* × *Node* | *e* ∈ *edges* • *e*. 1 = *n* ∨ *e*. 2 = *n*∀*e: Node* × *Node* | *e* ∈ *edges* • ∃*n1, n2: Node* | *n1* ∈ *nodes* ∧ *n2* ∈ *nodes* • *e* = (*n1, n2*)∀*n1, n2: Node* | (*n1, n2*) ∈ *edges* • (*n2, n1*) ∈ *edges*

#### Invariants

(1) For every node in the network topology, there is an ordered pair (edge) such that the node is either first or the second element of the ordered pair. (2) For every edge in the network, there exist two nodes such that the nodes formulate a link called edge in the network topology. It is assumed that the topology is a connected graph, that is, for any two nodes there is an edge. Any isolated actor in the network is not a part of the network topology.

Wireless sensor actor networks employ a number of sensor nodes that report an event of interest to one or multiple actors. The sensors are inexpensive and have scarce resources, whereas, actors are more powerful nodes in terms of energy, communication and computation power.

Formal specification of the sensor is described below by the schema Sensor. The schema contains five variables, namely, sensor identifier, its state, information about any event, neighbors and connectivity which are denoted by *id, state, information, neighbors* and *connectivity*, respectively. The state is a two-valued variable used to check if any event is captured. In the *information* variable, detail of the event is stored. As the sensor can communicate only with its neighbors, hence, information about neighbors is stored in the *neighbors* variable. The *connectivity* variable is used to check the status of the node which is either connected or disconnected to the network.

[*Data*]; *S* == *Node**State* ::= *OK* | *NOTOK**Connectivity* ::= *CONNECTED* | *DISCONNECTED* *Sensor**id: S**state: State**information:*

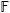

*Data**neighbours:*

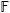

*Node**connectivity: Connectivity**state* = *OK* ⇔ *information* = ∅ ∧ *state* = *NOTOK* ⇔ *information* ≠ ∅*connectivity* = *CONNECTED* ⇔ *neighbours* ≠ ∅ ∧ *connectivity*= *DISCONNECTED* ⇔ *neighbours* = ∅

#### Invariants

(1) The sensor is responsible for continuous monitoring of the environment. If an unwanted object is detected the data will be collected by the sensor and stored in the *information* variable. In this property, it is stated that if state of sensor is normal (*OK*) then there is no information in the sensor. If state of sensor is not normal then there must be some information stored in the sensor. (2) As the wireless sensor and actor network is a distributed system, hence, it is assumed that every node will have information about its neighbours at 1-hop. In this property, it is stated that a node is connected if the collection of its neighbours is a non-empty set. The node is disconnected if the set of its neighbours is an empty set.

The role of an actor is extremely crucial for a timely response in order to prevent any serious consequences after a sensor node has reported an event. For this purpose, actors need to coordinate with each other for an effective and optimal response synchronizing the required operations. Therefore, actors establish and maintain inter-actor network topology in order to enable such communication. As the failure of a critical actor may partition the inter-actor network into a disjoint sub-network, consequently, backup preferably non-critical to every such node is required for the failure recovery. The backup actor node continuously monitors its critical, if any failure is detected then it initiates the recovery process by moving towards the primary until connectivity is restored. Formal specification of the actor is described below by the schema *Actor*. Based on the functionality, eight variables are extracted to describe role of an actor. As each actor must be uniquely identified like a sensor, hence, the first variable is the actor identifier. The second variable is used to define the type of the actor node and has two values, *i.e.*, either critical or non-critical. As in case of failure of a critical node, the backup is required, the third variable is used for this purpose. Of course the backup must be from its neighbors. The fourth variable is for storing information about neighbors, because an actor must know about its neighbors for a failure recovery. The *connectivity* variable is to check if the actor is itself connected or disconnected. The last three variables are used to store information about the reported events from the sensors. The *detected* variable is used for storing information about all reported events. The *pending* variable is used to record events for which the action is still required. And the last one variable *completed* is used for recording the information about events for which action is completed.

*A* == *Node**Criticality* ::= *CRITICAL* | *NONCRITICAL* *Actor**id: A**type: Criticality**backup: Neighbour**neighbours:*

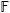

*Neighbour**connectivity: Connectivity**detected:*

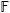

*Detected**pending:*

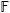

*Detected**completed:*

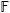

*Detected**type* = *CRITICAL* ⇒ # *neighbours* ≤ 2 ∧ *type* = *NONCRITICAL* ⇒ # *neighbours* > 2*backup . type* = *ACTOR*∃*nbr: Neighbour* | *nbr* ∈ *neighbours* • *nbr . neighbour* =*backup . neighbour*∀*nbr: Neighbour* | *nbr* ∈ *neighbours* ∧ *nbr . neighbours* ∈ 

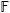

*Node*  ∧ *backup . neighbours* ∈ 

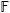

*Node* • # *backup . neighbours* ≤ # *nbr . neighbours**connectivity* = *CONNECTED* ⇔ *neighbours* ≠ ∅ ∧ *connectivity*= *DISCONNECTED* ⇔ *neighbours* = ∅∀*d: Detected* | *d* ∈ *pending* • *d . action* = *PENDING* ∧ *d . data*≠ ∅∀*d: Detected* | *d* ∈ *completed* • *d . action* = *COMPLETED* ∧ *d . data* = ∅∀*d: Detected* | *d* ∈ *detected* • *d* ∈ *pending* ∨ *d* ∈ *completed*∀*d: Detected* | *d* ∈ *pending* ∨ *d* ∈ *completed* • *d* ∈ *detected*

#### Invariants

(1) The node is critical if it has, at most, two neighbors and after its removal the network is partitioned, otherwise, the node is non-critical. (2) The backup of the actor node must be an actor node. (3) The backup of an actor node must be among one of its neighbours. (4) The backup of the node is the one with least degree among its neighbours. (5) The sensor node is connected if it has, at least one neighbour, otherwise it is disconnected. (6) As a safety property, in the set of detected sensors, if the action is pending then the detected issue exists in the database of the sensor. (7) In the set of detected sensors, if the action is completed then information is removed from the database of the sensor to make an optimized usage of the storage. (8) For each detected sensor, the action is either completed or pending. (9) For each sensor, if it is in the list of pending or completed then it must be in the list of detected sensors. The properties 8 and 9 are described for the consistency.

The schema *Detected* defined below is used to store the information about the reported events from any sensor to an actor. The schema consists of three variables, namely, reporting sensor identifier, the event details and action having two values, that is, completed or required.

*Action* ::= *PENDING* | *COMPLETED* *Detected**sensor: Node**data:*

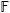

*Data**action: Action*

In case of sensor definition, only neighbor identifiers were defined, but in case of actors, more information about neighbors is required. This is because a sensor needs only to sense the event and report to the responsible actor(s). However, the actor is assumed more powerful and its job is to coordinate with other actors to complete the activity efficiently, accurately and effectively after receiving the information from the sensors. That is why it needs more information about its neighbors. The schema *Neighbour* (used in definition of actor) consists of five variables, namely, *neighbour* (identifier), *neighbours, type, criticality* and *distance*. The variable *criticality* is used because non-critical backup is preferred and, hence, this information is required for backup identification and assigning. The distance variable is used as it is also required for backup assigning and analysis.

*Type* ::= *ACTOR* | *SENSOR* *Neighbour**neighbour: Node**neighbours:*

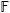

*Node**type: Type**criticality: Criticality**distance:* ℕ

As wireless sensor actor networks employs a number of sensors that report to actors and the concerned actors respond to events by following the network topology in order to prevent any serious consequences. The network is denoted by the schema *WSAN* which consists of three components, that is, *sensors, actors* and *topology*. The *sensors* is a power set of *Sensor* and similarly *actors* is a power set of *Actor*. The definitions of sensor, actor and topology are already described by using the schemas *Sensor, Actor* and *Topology*, respectively. All of these three main components of the network are put in first part of the schema and invariants are defined in second part of the schema in terms of predicates for well definedness.

 *WSAN**Topology**sensors:*

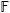

*Sensor**actors:*

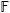

*Actor*∀*s: Sensor* | *s* ∈ *sensors* • *s . id* ∈ *nodes*∀*n: Node* | *n* ∈ *nodes* • (∃*s: Sensor* | *s* ∈ *sensors* • *n* = *s . id*) ∨(∃*a: Actor* | *a* ∈ *actors* • *n* = *a . id*∀*s: Sensor* | *s* ∈ *sensors* • ∀*nbr: Node* | *nbr* ∈ *s . neighbours* • (*s . id, nbr*) ∈ *edges*∀*a: Actor* | *a* ∈ *actors* • *a . id* ∈ *nodes*∀*a: Actor* | *a* ∈ *actors* • (*a . id, a . backup . neighbour*) ∉ *edges*∀*a: Actor* | *a* ∈ *actors* • ∀*nbr: Neighbour* | *nbr* ∈ *a . neighbours*  • (*a . id, nbr . neighbour*) ∈ *edges*∀*actor: Actor* | *actor* ∈ *actors* • ∃*sensor: Sensor* | *sensor* ∈ *sensors* • *actor . id* = *sensor . id*∀*s: Sensor* | *s* ∈ *sensors* • ∃*a: Actor; path:* seq *Node* | *a* ∈ *actors* ∧ ran *path* ⊆ *nodes*  • ∀*i:* ℕ | *i* ∈ dom *path* ∧ *i* ∈ 1 ‥ # *path* - 1 • *path* 1 = *s . id*   ∧ *path* (# *path*) = *a . id* ∧ (*path i, path* (*i* + 1)) ∈ *edges*

#### Invariants

(1) Every sensor of the WSAN is a node in the topology. (2) Every node in the topology is either a sensor or an actor of the WSAN. (3) Every neighbour of every sensor is connected in the topology. (4) Every actor of the WSAN is also a node in the topology. (5) Any neighbour of the backup of an actor is not connected directly to the actor. (6) Every neighbour of every actor is connected with the actor in the topology. (7) For every actor there is a sensor having same identifier, that is, every actor acts as a sensor as well. (8) For every sensor there is a path from the sensor itself to some of the actors in the network. That is for every sensor there is, at least, one actor responsible for receiving information and performing an action. The path is defined as a sequence of nodes. The first element in the sequence is sensor and the last element is actor. Further, for every pair of consecutive elements in the sequence, it is an ordered pair and an edge in the network topology.

As we know, failure of a critical actor divides the network into disjoint sub-networks. Hence, identification of critical actors is important after deployment of the network. Formal specification of identifying critical nodes is given below using the schema *CriticalsIdentification*. The schema takes *WSAN* as input and returns the set of critical nodes as output by using the *criticals!* variable. In Z notation, the symbol “!” is used after end of the output variable. As defined in the informal description of PCR algorithm, it employs a simple localized cut-vertex detection procedure that only requires 1-hop positional information to detect critical nodes. The 1-hop information is stored in terms of neighbors of any node. The formal description of the critical nodes identification procedure is given in second part of the schema and its informal description is provided in terms of properties following the schema.

 *CriticalsIdentification*Ξ*WSAN**criticals!:*

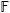

*Actor*∀*a: Actor* | *a* ∈ *criticals!* • *a* ∈ *actors**criticals!* = {*a: Actor* | *a* ∈ *actors* ∧ (∃*sa, sb:*

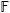

*Node* | *sa* ⊆ *nodes* \ {*a . id*} ∧ *sb* ⊆ *nodes* \ {*a . id*} • (*sa* ∩ *sb* = { }∧ (∀*n1, n2: Node* | *n1* ∈ *sa* ∧ *n2* ∈ *sb*  • (∀*path:* seq *Node* | ran *path* ⊆ *nodes* \ {*a . id*}  • (∀*i:* ℕ | *i* ∈ dom *path* ∧ *i* ∈ 1 ‥ # *path* - 1  • (*path* 1 ≠ *n1* ∨ *path* (# *path*) ≠ *n2* ∨ (*path i, path* (*i* + 1) ∉ *edges* \ {*n: Node*  | *n* ∈ *nodes* \ {*a . id*} • (*a . id, n*)})))))) • *a*}

#### Properties

(1) Every critical node must be an element of the set of actors of the WSAN. (2) The set of critical actors of WSAN is identified and calculated as follows: if the critical node is removed from the network then the network is divided into at least two disjoint sub-networks. That is after removal of critical node from the network there exists at least two sets A and B of nodes such that A ∩ B = ∅ and there does not exist any path from some nodes of A to some nodes of B. Hence if the network is partitioned after removal of a node from the network then the node is identified as critical and put into the set of critical nodes.

Once the critical actors are identified, it needs to select and designate an appropriate neighbor to each critical as a backup. The formal specification of the criteria assigning a backup node is given below using schema *BackupAssigning*. The selection of backup is based on the following procedure: it is determined that the backup is critical or non-critical. A non-critical actor is preferred to serve as backup. A non-critical actor with the least degree is a suitable candidate. The close backup actor is preferred in order to reduce the movement overhead and shorten the recovery time.

  *BackupsAssigning* Δ*WSA**CriticalsIdentification* ∀*a: Actor* | *a* ∈ *criticals!* • *(*∃*nbr: Neighbour*  | *nbr* ∈ *a . neighbours* ∧ *nbr . criticality* = *NONCRITICAL*  ∧ *(*∀*nbr1: Neighbour* | *nbr1* ∈ *a . neighbours*  ∧ *nbr1* ≠ *nbr* ∧ *nbr . neighbours* ∈ 

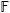

*Node* ∧ *nbr1 . neighbours* ∈ 

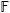

*Node*   • # *nbr . neighbours* ≤ # *nbr1 . neighbours)* • *a . backup* = *nbr)*  ∨ *(*∃*nbr1: Neighbour* | *nbr1* ∈ *a . neighbours*  ∧ *(*∀*nbr2: Neighbour* | *nbr2* ∈ *a . neighbours* ∧ *nbr2* ≠ *nbr1*   • *nbr1 . distance* ≤ *nbr2 . distance)* • *a . backup* = *nbr1)*

#### Properties

For every critical actor of WSAN as identified by the above schema, the backups are assigned by the following criteria: backup must be preferably non-critical, has the least degree among its neighbours and, has least distance as compared to all other backup candidates.

In the above schemas, a formal procedure of identifying the critical nodes is defined and then a criteria of assigning a backup to the critical node is described. Now formal specification of assigning backups to the critical nodes is described below using the schema *CriticalsBackups*. The schema takes *WSAN* as input and assigns the backups to the critical nodes using the variable *cribackups!*. At first the critical node is verified and then an appropriate neighbor to the critical node is assigned as a backup meeting the required criteria. In the backup assigning, initially, the backup neighbor is preferred to be non-critical. Then least degree and close distance properties are verified as preferred requirements. If no such backup exists, then the condition of non-criticality is relaxed, however, the other conditions of assigning the backup must be satisfied.

 *CriticalsBackups*Ξ*WSAN**cribackups!:*

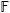

*Actor*∀*a: Actor* | *a* ∈ *cribackups!* • *a* ∈ *actors**cribackups!* = { *a: Actor* | *a* ∈ *actors*  ∧ (∃*sa, sb:*

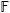

*Node* | *sa* ⊆ *nodes* \ {*a .id*} ∧ *sb* ⊆ *nodes* \ {*a .id*}   • (*sa* ∩ *sb* = { } ∧ (∀*n1, n2: Node* | *n1* ∈ *sa* ∧ *n2* ∈ *sb*   • (∀*path:* seq *Node* | ran *path* ⊆ *nodes* \ {*a . id*}   • (∀*i:* ℕ | *i* ∈ dom *path* ∧ *i* ∈ 1 ‥ # *path* - 1 • (*path* 1 ≠ *n1*  ∨ *path* (# *path*) ≠ *n2* ∨ (*path i, path* (*i* + 1)) ∉ *edges* \ {*n:*   *Node* | *n* ∈ *nodes* \ {*a . id*} • (*a . id, n*)}))))))  ∧ (∃*nbr: Neighbour* | *nbr* ∈ *a . neighbours*  ∧ *nbr . criticality* = *NONCRITICAL* ∧ (∀*nbr1: Neighbour*   | *nbr1* ∈ *a . neighbours* ∧ *nbr1* ≠ *nbr*  ∧ *nbr . neighbours* ∈ 

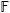

*Node* ∧ *nbr1 . neighbours* ∈ 

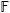

*Node*   •# *nbr.neighbours*≤# *nbr1.neighbours*) • *a .backup* = *nbr*)  ∨ (∃*nbr1: Neighbour* | *nbr1* ∈ *a . neighbours*  ∧ (∀*nbr2: Neighbour* |*nbr2* ∈ *a .neighbours* ∧ *nbr2* ≠ *nbr1*   • *nbr1.distance* ≤ *nbr2.distance*) • *a.backup* = *nbr1*) • *a*}

#### Properties

(1) Every critical node for which the backup is required, must be an element of the set of actors of the network. (2) In this property, first of all, it is verified that the node is critical for which backup is required by using the same logic as used in identification of critical nodes. Then existence of non-critical node among the neighbors is checked for assigning the backup, and if such a node exists it is assigned as a backup preferably with least degree and least distance for movement in case of recovery of the network. If there does not exist a non-critical node in the neighbors then a critical node might be assigned by meeting the other two conditions of least degree and least distance. If a non-critical node does not exit and there are more than one critical nodes with the same degrees of incidence then the node with minimum distance of movement will be selected as a backup node. In the property, the same process is repeated and defined for all the critical nodes for assigning the backups.

Formal specification of verifying a failure node is described below by the schema *Failure* in the network topology. The node to be verified as failure is given as input and is checked if it is connected in the network topology. The symbol “?” at the end of *failure?* variable shows that it is an input to the schema *Failure*. This is a common practice in Z notation in defining input to any operation. The symbol Δ used before WSAN in the schema shows that state of the schema is changed.

 *Failure*Δ*WSAN**failure?: Actor**failure?* ∈ *actors**failure? . neighbours* = { }*failure? . connectivity* = *DISCONNECTED**nodes'* = *nodes* \ {*failure? . id*}*edges'* = *edges* \ {*n: Node* | *n* ∈ *nodes* ∧ (*failure? . id, n*) ∈     *edges* • (*failure? . id, n*) }*actors'* = *actors* \ {*failure?*}∃*s: Sensor* | *s* ∈ *sensors* • ∀ *a: Actor* | *a* ∈ *actors* • ∀*path:* seq *Node* | ran *path* ⊆ *nodes*  • ∀*i:* ℕ | *i* ∈ dom *path* ∧ *i* ∈ 1 ‥ # *path* - 1   • *path* 1 ≠ *s . id* ∨ *path* (# *path*) ≠ *a . id*    ∨ (*path i, path* (*i* + 1)) ∉ *edges*

#### Properties

(1) The failure node is an element of the set of critical nodes. (2) There does not exist any neighbor of failure, that is, the node is disconnected from the network. (3) The status of the failure node is disconnected in the connectivity variable. (4) The network topology is updated by removing the node from the set of nodes. (5) The network is disconnected and updated by removing all the edges incident at the failure node. (6) The set of actors is also updated in the network. (7) Finally, it is verified that there exist some sensors which cannot communicate with any of the actors to prove that network is disconnected.

Once the failure node is detected and verified, the recovery process is initiated. The formal specification of the failure recovery is described by the *Recovery* schema. At each step of movement of a node few links will be removed and some others will be added. In formal specification, four variables are defined in addition to WSAN schema. The first one *failure*? variable represents the failure node which will be replaced with a new node named as *replaced!*. The other two variables represent removed and added links after movement of a node from its own position to replace the neighbor.

 *Recovery*Δ*WSAN**failure?, replaced!: Actor**brokenlinks:*

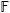
 (*Node* × *Node*)*newlinks:*

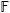
 (*Node* × *Node*)*failure?* ∈ *actors**replaced!* ∈ *actors* \ {*failure?*}*replaced!* = *failure?**actors'* = *actors* \ {*failure?*} ∧ *sensors'* = *sensors**nodes'* = *nodes* \ {*failure? . id*}*edges'* = *edges* \ *brokenlinks* ∪ *newlinks*∀*s: Sensor* | *s* ∈ *sensors'* • ∃*a: Actor; path:* seq *Node* | *a* ∈ *actors'* ∧ ran *path* ⊆ *nodes'*  • ∀*i:* ℕ | *i* ∈ dom *path* ∧ *i* ∈ 1 ‥ # *path* - 1 • *path* 1 = *s . id*   ∧ *path* (# *path*) = *a . id* ∧ (*path i, path* (*i* + 1)) ∈ *edges'*

#### Invariants

(1) The failure node exists in the set of nodes of the old topology. (2) The replaced node is not the failure node and is an element of the set of nodes of new topology. (3) The position of failure node is replaced with the newly replaced node. (4) The state of actors is updated after removing failure node from the collection of old actor-set. (5) The state of set of sensors is not changed. (6) The set of nodes of the topology is also updated after removing the failure node from the collection of old node-set. (7) The communication links of the network topology are also updated by removing the broken links and adding the newly established links. (8) Finally, it is verified that the network is connected, that is, for every sensor there is, at least, one actor such that there exists a path from the sensor to the actor in the network topology.

### 5.5. Model Analysis Using Z/Eves

In this section, model analysis is done for the specification. It is noted that although computer tools improve the quality of software systems, on the hand, there does not exist any real computer tool which can ensure complete correctness of a model. It means even if the specification is well-written using any of the specification languages, it may still contain potential errors. That is an art of writing a formal specification that never guarantees that the system is correct, complete and consistent. However, if the specification is analyzed with computer tools it increases a confidence over the system to be developed.

**Figure 7.**
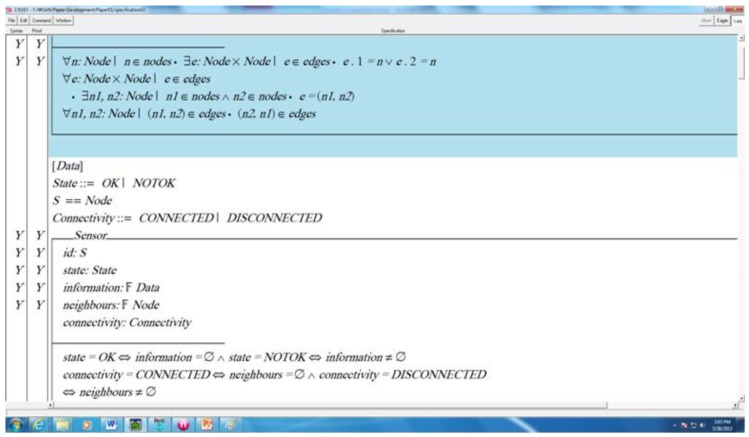
Snapshot of the model analysis.

The Z/Eves is one of the powerful tools used for the analysis of Z specifications which is applied to analyze the system's schema expansion, pre-condition calculation, domain checking, syntax and type checking, and theorem proving. This is why we have used Z/Eves for analyzing the Z specification of hybrid connectivity restoration algorithm for WSAN. A snapshot of the specification analysis is presented in Figure 7. The first column on the left of the figure shows syntax checking and the second column represents the proof correctness of the specification. The symbol ‘Y’ shows that the formal specification is syntactically correct and proof is also correct, while the symbol ‘N’ represents that errors exist. All the schemas are checked to prove that specification is correct in syntax and has a correct proof. Some schemas of the specification were proved using reduction techniques available in the toolset.

Summary of the analysis is presented in Table 1. In first column of the table, name of schema is given. The symbol “Y”, in column 2, indicates that all schemas are well-written and proved. Similarly, domain checking, reduction and proof by reduction are represented in columns 3, 4 and 5, respectively. The character “Y*” annotated with ‘*’ shows that the schema is proved by performing reduction on the predicates part to make specification more meaningful.

## 6. Results and Analysis

In addition to specification analysis, we validated the effectiveness of PCR through extensive simulations. This section describes the simulation environment, performance metrics and experimental results.

### 6.1. Experiment Setup and Performance Metrics

In the simulation experiments, we have created connected inter-actor topologies that contain varying numbers of nodes (20–100). Nodes are deployed randomly in an obstacle-free area of 1,000 m × 600 m. To observe the impact, we have changed the transmission range of actors (50–200). The following performance metrics were used to assess the performance:

- The *total distance moved* by all nodes involved in the recovery: This reflects the efficiency of PCR in terms of energy efficiency and movement overhead incurred during recovery.
- The *number of nodes* engaged during the recovery: This metric indicates the scope of the recovery process.
- The *number of messages* required to coordinate recovery among nodes: Again this metric gauges the communication overhead during recovery and ultimately affects energy dissipation.
- The *percentage of coverage reduction* relative to the pre-failure level: Although the prime concern of PCR is to restore connectivity, however, node coverage is important for many setups. In addition to coverage loss due to node failure, the recovery process may negatively impact coverage. This metric assesses the effectiveness of PCR in terms of coverage reduction.
- The *degree of connectivity* maintained after recovery: This metric indicates the availability of node independent paths and is measured by averaging the number of neighbors for each node.

The following parameters were used to vary the WSAN configuration in the experiments:

- The *number of actor nodes (N)* in the network affects the node density and the inter-actor connectivity.
- The *node communication range (r)* influences the network connectivity and highly affects the recovery overhead in terms of movement distance and scope of recovery.

### 6.2. Baseline Approaches

We compare the performance of PCR to the baseline approaches DARA [3], RIM [13] and DCR [27]. Like PCR, baseline approaches are distributed and exploit node mobility to restore connectivity. However, their procedures are different. When a node *F* fails, DARA selects a best candidate *A* among its 1-hop neighbors and replaces it. The algorithm is recursively applied to tolerate connectivity loss due to movement, *i.e., A* will be replaced with one of its neighbors and so on. On the other hand, RIM moves all the 1-hop neighbors towards *F* until they become connected. Like DARA, RIM is applied recursively to re-establish links affected by nodes movement. Both DARA and RIM are reactive schemes and do not provide for recovery ahead of time. Like PCR, DCR is a hybrid scheme that assigns a highest degree node as a backup.

### 6.3. Results and Analysis

The simulation experiments involve randomly generated topologies with varying actor counts and communication ranges. The number of actors has been set to 20, 40, 60, 80 and 100. The communication range of actors is changed among 50, 100, 150 and 200. When changing the node count, “*r*” is fixed at 100 m; and “*N*” is set to 60 while varying the communication range. Like baseline approaches, PCR is meant to recover from single actor failure. PCR may handle multiple node failures if they are far apart from each other and cause no conflicts during recovery. However, PCR and baseline approaches for a single node failure are not guaranteed to converge. Simultaneous failure of multiple nodes may cause conflicting conditions for PCR to converge successfully. Generally, the possibility of multiple simultaneous actor failure is exceptional; however, it may precipitated by disastrous events such as explosions in a battlefield. In such a case, failed actors are usually co-located. Recovery from such failures is very challenging and requires careful consideration. To handle a special case of multi-node failure, we have presented RAM in [27]. In the experiments, we identify critical actors and choose a cut-vertex at random to be failed. The results of individual experiments are averaged over 30 trials to ensure statistically stable results. All results are subject to 90% confidence interval analysis and stays within 10% the sample mean.

#### Total distance moved

**Figure 8.**
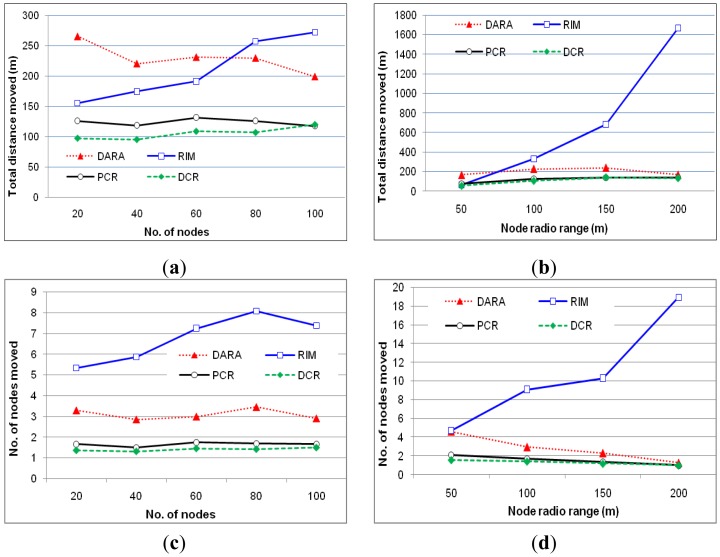

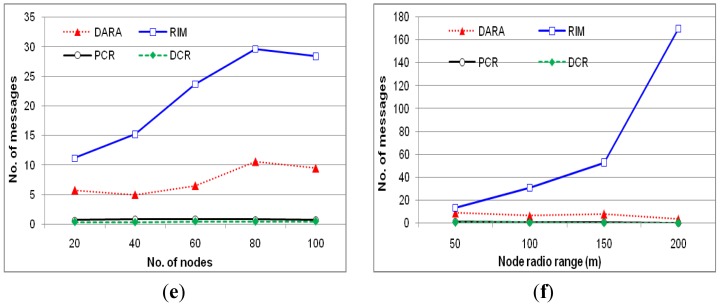
The total movement distance as a function of *N* in (**a**) and *r* in (**b**). The effect of changing *N* (**c**) and *r* (**d**) on number of nodes moved. Number of coordination messages, as a function of *N* in (**e**) and *r* in (**f**).

Figure 8(a,b) show the total distance traveled by nodes involved in recovery. PCR significantly outperforms reactive schemes, *i.e.*, DARA and RIM, because it strives to move non-critical nodes in order to prevent successive relocations. Both the graphs suggest that the performance advantage of PCR remains almost consistent while varying *N* and *r*. This is because PCR strives to avoid moving critical nodes that causes further partitioning and requires cascaded relocations. Furthermore, PCR only performs cascaded relocations in case non-critical backup in the neighborhood is not available. Figure 8(a) indicates that the performance of PCR scales very well and is not affected by the node density because of choosing non-critical nodes as backup. Similar observation can be made for the communication range as in Figure 8(b), where the movement overhead is significantly less as compared to the reactive schemes. However, PCR slightly performs worse than DCR. This is because DCR prefers to engage strongly connected nodes as backup. The rationale is that there is more probability to have non-critical nodes in the neighborhood of highly connected nodes.

#### Number of moved nodes

Figure 8(c,d) report the scope of recovery when PCR and the baseline approaches are applied. The graphs confirm the performance advantage of PCR which moves fewer actors than reactive schemes. This is because PCR limits the scope of recovery and avoids successive cascaded relocations by choosing non-critical nodes as backup. Furthermore, the performance of PCR remains almost constant while varying *N* and *r*, which indicates great scalability. However, again DCR slightly moves less number of nodes than PCR due to the same reason mentioned above.

#### Number of messages exchanged

Figure 8(e,f) show the coordination messaging overhead as a function of the *N* and *r*. As expected, hybrid schemes (PCR and DCR) cause far less coordination overhead than DARA and RIM. This is because they limit message exchange only between a pair of primary and backup instead of all 1-hop and 2-hop neighbors as is the case in RIM and DARA respectively. In addition, unlike reactive schemes, PCR and DCR strive to engage non-critical nodes in the recovery which does not require coordination messages. Figure 8(e) shows that the messaging overhead of reactive schemes increases with the high node density. This is because the number of 1-hop and 2-hop neighbors increases. Moreover, Figure 8(e,f) indicate that the messaging overhead in RIM significantly grows for a high actor density and long communication range because the number of recovery participants increases in both cases. On the other hand, the performance gap between PCR and DCR is minor relative to DARA and RIM.

#### Percentage of coverage reduction

**Figure 9.**
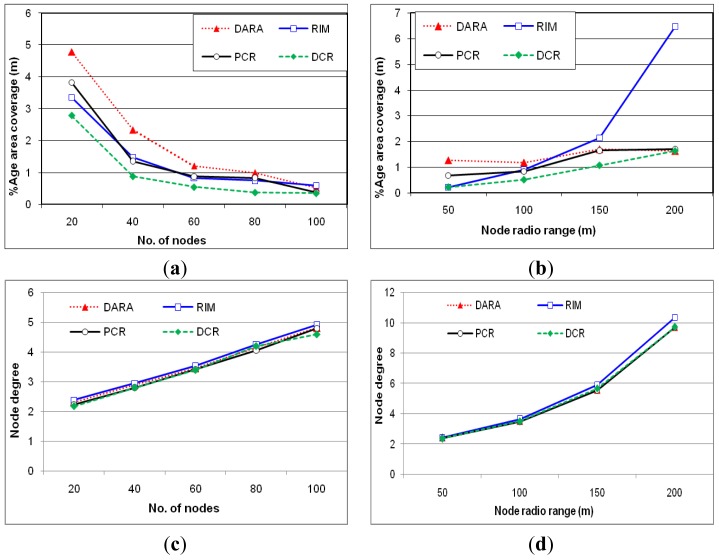
The coverage reduction after recovery, as a function of *N* in (**a**) and *r* in (**b**). Degree of connectivity after recovery, while varying the network size (**c**) and radio range (**d**).

Figure 9(a,b) report on the impact of recovery on coverage, measured in terms of percentage of coverage reduction relative to the pre-failure level, while changing the *N* and *r*. The action range is set to 50 m throughout these experiments. Overall, DCR confines the coverage loss and consistently outperforms other schemes. This result is attributed to the fact that moving highest degree nodes reduces overlapped coverage with neighbors and occupies the vacant spot. Figure 9(a) demonstrates that increasing the node density helps, other schemes in mitigating the coverage loss. The limited scale relocation slightly helps PCR to minimize loss of coverage. Figure 9(b) indicates that the performance of RIM significantly worsens when growing the communication range. This is because RIM collectively engages neighbors in recovery and with the increased value of *r*, the network becomes more connected and the number of neighbors of *F* grows. RIM moves nodes inwards making the area around *F* to be more crowded (as we shall see later) while leaving uncovered parts at the network periphery and thus causes a significant loss of coverage. On the other hand, the schemes that employ a node in recovery among neighbors is not much affected while increasing the communication range.

#### Degree of connectivity

Figure 9(c,d) demonstrate the degree of connectivity preserved by all approaches after recovery. Both figures clearly indicate that PCR maintains the same degree of connectivity as the other approaches despite several other performance advantages. This is mainly because PCR prefers to move non-critical and/or least degree nodes that have nominal repercussions on the connectivity.

## 7. Conclusion and Future Work

This paper has presented a hybrid partitioning detection and connectivity restoration algorithm for mission-critical applications of WSANs. Unlike most published schemes, PCR pursues pre-failure planning to distinguish between critical/non-critical nodes and designates an appropriate backup for each critical actor. The pre-designated backup continuously monitors and triggers a recovery in case of primary failure. The correctness and performance of the proposed scheme is validated through formal and simulation techniques respectively. We modeled the WSAN network as a dynamic graph and transformed PCR into a corresponding formal specification using Z notation. This formal specification is analyzed and validated using the Z Eves tool. Moreover, the performance of PCR is validated through extensive simulations. Simulation results have confirmed the effectiveness of PCR compared to contemporary schemes found in literature. In the future, we plan to evaluate the performance of PCR through a testbed network consisting of mobile robots.

## Figures and Tables

**Table 1.** Results of model analysis.

**Schema Name**	**Syntax Type Check**	**Domain Check**	**Reduction**	**Proof**
*Topology*	Y	Y	Y	Y
*Sensor*	Y	Y	Y	Y
*Detected*	Y	Y	Y	Y
*Neighbour*	Y	Y	Y	Y
*Actor*	Y	Y	Y*	Y
*WSAN*	Y	Y	Y*	Y
*CriticalsIdentification*	Y	Y	Y*	Y
*BackupsAssigning*	Y	Y	Y*	Y
*CriticalsBackups*	Y	Y	Y*	Y
*Failure*	Y	Y	Y*	Y
*Recovery*	Y	Y	Y*	Y
